# Human Placenta and Markers of Heavy Metals Exposure

**DOI:** 10.1289/ehp.1206061

**Published:** 2013-01-01

**Authors:** Paolo D. Pigatto, Claudio Minoia, Anna Ronchi, Gianpaolo Guzzi

**Affiliations:** 1 Department of Bioscience for Health, Dermatological Clinic, IRCCS Galeazzi Hospital, University of Milan, Milan, Italy; 2 Laboratory of Environmental and Toxicology Testing, Fondazione Salvatore Maugeri, IRCCS (Istituto Di Ricovero e Cura a Carattere Scientifico), Pavia, Italy; 3 Italian Association for Metals and Biocompatibility Research (AIRMEB) Milan, Italy, E-mail: gianpaolo_guzzi@fastwebnet.it

In their review, Esteban-Vasallo et al. (2012) discussed the use of human placenta to evaluate biomarkers of exposure to heavy metals. They correctly concluded that the use of placental tissue specimens to assess heavy metal exposure is actually underused. Surprisingly, they did not mention the well-documented relationship between mercury released from mercury-containing dental amalgam fillings and mercury disposition in placental tissues (Clarkson and Magos 2006; Gundacker and Hengstschläger 2012; Richardson et al. 2011).

Studies have suggested an association between mercury levels in placental tissues and the observed mercury dental amalgams in women (Ask et al. 2002; Palkovicova et al. 2008; Richardson et al. 2011). Elevated placental mercury levels have been reported in dental workers who, throughout pregnancy, were exposed to mercury vapor (Hg^0^) released during preparation of mercury amalgam in dental offices (Guzzi and Pigatto 2007; Wannag and Skjaeråsen 1975). As noted by Drasch et al. (1994), the mother-to-fetus transfer of mercury Hg^0^ from amalgams has been reported in human autopsy samples, and elevated levels of total mercury have been observed in the brain, liver, and kidney of human fetuses; these levels have been linked to the number of maternal amalgam-restored surfaces.

Transplacental exposure to heavy metals may affect child growth and cause neurodevelopmental delays. Thus, further efforts should be made to measure and quantify maternal exposure to heavy metals in placenta to estimate environmental prenatal exposure.
